# Surgical technique and clinical results for scapular allograft reconstruction following resection of scapular tumors

**DOI:** 10.1186/1756-9966-28-45

**Published:** 2009-04-01

**Authors:** Kaiwei Zhang, Hong Duan, Zhou Xiang, Chongqi Tu

**Affiliations:** 1 Department of Orthopedics, West China Hospital, Sichuan University, Chengdu, 610041, PR China

## Abstract

**Background:**

Progress in developing effective surgical techniques, such as scapular allograft reconstruction, enhance shoulder stability and extremity function, in patients following scapular tumor resection.

**Methods:**

Case details from seven patients who underwent scapular allograft reconstruction following scapular tumor resection were reviewed. A wide marginal resection (partial scapulectomy) was performed in all patients and all affected soft tissues were resected to achieve a clean surgical margin. The glenoid-resected and glenoid-saved reconstructions were performed in three and four patients, respectively. The residual host scapula were fixed to the size-matched scapular allografts with plates and screws. The rotator cuff was affected frequently and was mostly resected. The deltoid and articular capsule were infrequently involved, but reconstructed preferentially. The remaining muscles were reattached to the allografts.

**Results:**

The median follow-up was 26 months (range, 14–50 months). The average function scores were 24 points (80%) according to the International Society of Limb Salvage criteria. The range of active shoulder abduction and forward flexion motion were 40°–110° and 30°–90°, respectively. There was no difference between the glenoid-saved and glenoid-resected reconstructions in the total scores (mean, 24.5 points/81% versus 24 points/79%), but the glenoid-saved procedure was superior to the later in terms of abduction/flexion motion (mean, 72°/61° versus 55°/43°). During the study follow-up period, one patient died following a relapse, one patient lived despite of local recurrence, and five patients survived with no evidence of recurrence of the original cancer. Post-surgical complications such as shoulder dislocations, non-unions, and articular degeneration were not noted during this study period.

**Conclusion:**

Scapular allograft reconstruction had a satisfactory functional, cosmetic, and oncological outcome in this case series. Preservation and reconstruction of the articular capsule and deltoid are proposed to be a prerequisite for using scapular allografts and rotator cuff reconstruction is recommended, although technically challenging to perform.

## Background

For treatment of shoulder girdle tumors, scapulectomy and the Tikhoff-Linberg procedure were initially designed in an attempt to preserve hand and elbow performance. Unfortunately, functional impairment of the shoulder and the poor cosmetic outcome (e.g., flail arm) were widely described following these procedures. An array of other limb-sparing procedures for the treatment of shoulder girdle tumors have also been documented [1-11] with variable results in relation to shoulder function. With recent improvements in effective adjuvant therapy and surgical techniques, restoring shoulder stability, preserving a functional upper extremity, and rebuilding the shoulder contour after scapular tumor resection is feasible in many cases. Several reconstruction procedures for the scapula have been introduced over the last thirty years, including prosthesis or graft reconstruction of the shoulder girdle. Total scapular prosthesis has proven itself to be a safe and reliable method for reconstructing the shoulder girdle after resection of bony and soft tissue tumors of the scapula. Further, good to excellent shoulder function and cosmetics have been reported for scapular prosthesis [5-8]. The disadvantage of this procedure, however, is the insecure soft tissue reconstruction and the loss of the uninvolved proximal humerus.

Scapular reconstruction using allografts following resection of scapular tumors have rarely been reported. Nonetheless, osteoarticular acetabular allograft and scapular allograft reconstructions of the scapula have been described and are associated with a satisfactory functional and cosmetic result [2-4,12]; however, the surgical technique and related clinical results have not been presented in detail. Therefore, the purpose of this study was to highlight the issues surrounding scapular allograft reconstruction, including those associated with the incision, resection, surgical margin, and bone and soft tissue management, and to present the clinical results of this procedure in a series of seven patients.

## Methods

### Patients

Case details from seven patients (five males and two females) with scapular tumors who underwent scapular allograft reconstruction between 2004 and 2007 were reviewed. The average age of the patients was 37 years (range, 14–66 years). The diagnosis of every patient was established by preoperative biopsy. Five patients were diagnosed with primary malignant tumors of the scapula (two chondrosarcomas, one myeloma, one non-Hodgkin's lymphoma, and one osteosarcoma) whereas the remaining two patients were diagnosed with aggressive benign tumors (primary and recurrent chondroblastoma).

The one patient with recurrent chondrosarcoma (#2) underwent tumor resection of the proximal humerus eight months before presenting to our hospital. Another patient (#6), with recurrent chondroblastoma underwent partial scapulectomy at an outside institution two months before presenting to our hospital. The patient with osteosarcoma (#7) received neoadjuvant chemotherapy pre- and postsurgically. The patients with myeloma (#3) and malignant lymphoma (#4) received local intraoperative radiotherapy.

Data for all patients, including gender, age, diagnosis, cancer stage, adjuvant therapy, time of follow-up, and reconstruction type, have been summarized in Table 1. All patients were staged by local radiography, magnetic resonance imaging, computed tomography, nuclear scintigraphy (technetium bone scans), and preoperative biopsy. The resection and management of bone and muscle, together with the clinical results, have been provided in Table 2. Shoulder function was evaluated according to the International Society of Limb Salvage criteria (ISOLS) criteria [13], as shown in Table 3.

**Table 1:** Patient Data

**Patient**	**Gender/Age (years)**	**Diagnosis**	**Stage**	**Adjuvant therapy**	**Followup (months)**	**Glenoid-saved/resected reconsturction**
1	M/38	CS	IB	None	14	Glenoid-resected
2	M/29	RCS	IB	None	38	Glenoid-saved
3	M/66	ML	IB	I^125^	20	Glenoid-saved
4	F/29	NHL	IIB	I^125^	16	Glenoid-resected
5	M/46	CB	3	None	16	Glenoid-resected
6	F/40	RCB	3	None	29	Glenoid-saved
7	M/14	OS	IB	NAC	50	Glenoid-saved

CS: chondrosarcoma, RCS: recurrent chondrosarcoma, ML: myeloma, NHL: non-Hodgkin lymphoma, CB: chondroblastoma, RCB: recurrent chondroblastoma, OS: osteosarcoma, NAC: neoadjuvant chemotherapy.

**Table 2:** Resection and Reconstruction of Bone and Muscles and Clinical Results

**Patient**	**Muscles resected**	**Muscles reconstructed**	**Site of bony lesion**	**Size of lesion (cm^3^)**	**Oncological results**	**Complications**
1	SS, IS, SSC, BB, SA	SS, IS, BB	S, SF, AP, G, CP, SN	25 × 5 × 6	A	None
2	SS, IS, SSC, RR, LS, T	SS, IS, T, R	S, SF, AP, C, CP	10 × 8 × 7	A	Deep infection
3	T, TMI, TMA, SSC, LD	T, LD, SSC	SN, AP, S, G	16 × 11 × 4	AWD	None
4	SS, IS, SSC, BB, CB, TMI, TMA, D	D, CB, BB	SN, G, SF	12 × 11 × 10	A	Pain
5	SS, IS, SSC	SS, IS, SSC	S, SF	7 × 4 × 4	A	None
6	SS, IS, SSC, TB, TMI, TMA	None	S, SF, IF, TBS	5 × 4 × 3	DOD	Dead
7	SS, IS, SSC, T, D, TMI, TMA, LD, SA	T, LD	SF, G, SN, CP	20 × 15 × 5	A	None

SS: supraspinatus, IS: infraspinatus, TMI: teres minor, TMA: teres major, SSC: subscapularis, SA: serratusanterior, D: deltoid, BB: biceps brachii, TB: triceps brachii, T: trapezius, LD: Latissimus dorsi, R: rhomboideus, LS: levator scapula, CB: coracobrachialis, R: rhomboideus, SF: supraspinous fossa, IF: infraspinous fossa, S: spine, CP: coracoid process, AP: acromion process, SN: scapular neck, G: glenoid, C: clavicle, TBS: the border of the scapula, DOD: dead of disease, AWD: alive with disease, A: alive without evidence of disease.

**Table 3:** Functional Results According to ISOLS Criteria

**Case**	**Pain**	**Function**	**Emotional acceptance**	**Hand positioning**	**Manual dexterity**	**Lifting ability**	**Total score**	**Abduction and flexion**
1	5	3	3	3	5	3	22(73%)	50°-30°
2	5	4	5	5	5	4	28(93%)	110°-80°
3	5	3	5	4	5	4	26(86%)	80°–90°
4	3	3	4	5	5	3	23(76%)	35°–45°
5	5	4	5	5	5	3	27(90%)	80°-55°
6	5	2	3	3	5	3	21(70%)	40°-35°
7	5	3	4	4	4	3	23(76%)	60°-40°

### Surgical approach

**Figure 1:**
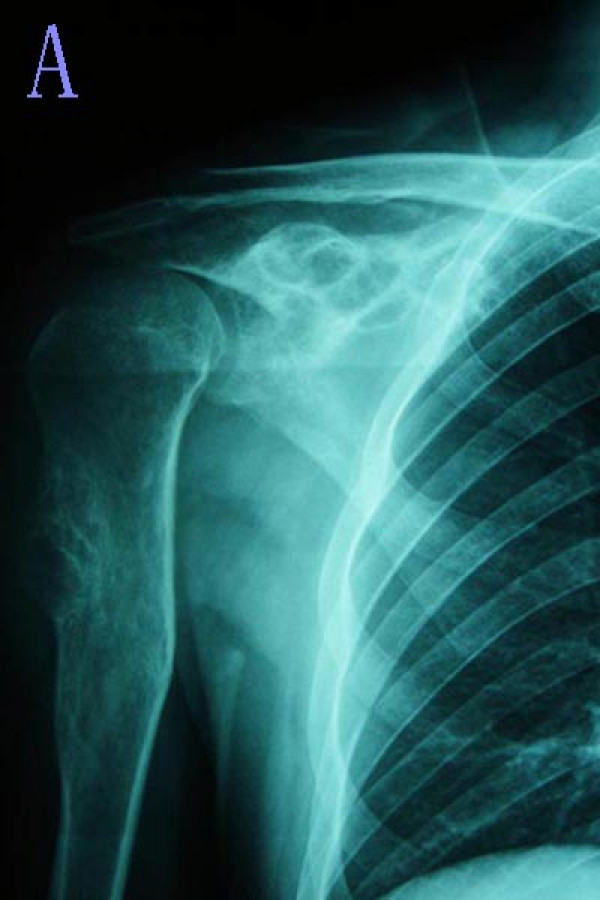
**Radiographs of the patient with primary chondrosarcoma (#1)**. **(A) **The plain radiograph shows a lytic bony lesion in S2. The other lesion in the proximal humerus was identified as chondroma.

**Figure 2:**
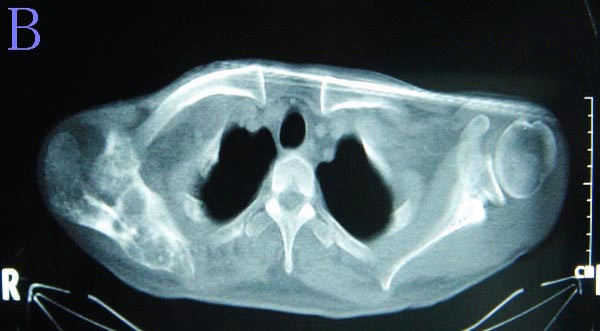
**Computed tomography scan shows the scapular lesion expanding into the surrounding muscles**.

The approach to the tumor for each patient was determined by precise preoperative imaging studies. The primary lesion of the scapula for all seven patients were mainly detected in region S2, the acromion/glenoid complex (Figure 1, Figure 2) with partial lesions occurring in region S1, the blade/spine of the scapula as categorized using the MSTS classification [1]. The incision was centered in the middle of the tumor. Thus, a posterior extensile incision was made in four patients (#1, 2, 5, and 6) starting at the inferior angle along the medial border of the scapula, curving laterally through the spine to the tip of the acromion. The overall length of the incision was determined based on the extent of each patient's lesion. In another patient (#7), a vertical incision was created that extended along the lateral border from the inferior angle of the scapula to the intermedial portion of the clavicle, following the previous incision made during a prior partial scapulectomy. In another patient, (#3) the incision had the same starting point as the patient #7, but then extended medially from the lateral superior angle to the medial superior angle of the scapula along the spine. In the last patient, (#4) the incision was extended from the sternoclavicular joint along the clavicle and continued over the shoulder along the deltopectoral groove.

### Resection and surgical margins

The affected supraspinatus, infraspinatus, and subscapularis were identified in six patients (#1, 2, 4, 5, 6, and 7). The involved teres minor and teres major in four patients (#3, 4, 6, and 7) and the affected trapezius in three patients (#2, 3, and 7) were identified. The involved partial deltoid (anterior or posterior), latissimus dorsi, and biceps brachii were identified in two patients, respectively (#4 and 7, #3 and 7, and #1 and 4). The affected serratus anterior, coracobrachialis, rhomboideus, and the suprascapularis were identified in one patient each (#1, 4, 2, and 1, respectively). The articular capsule was essentially intact in all patients. After exposing each patient's tumor, the supporting musculature was examined. The rotator cuff was involved most frequently (particularly the supraspinatus) and the deltoid was involved at a somewhat lower frequency.

A partial scapulectomy (Type IIA) was performed in all patients as previously described [14]. The resection of all involved soft tissues was extralesional, with a 2 to 5 cm margin. Thus, according to the extent of the lesion in these patients, little of the deltoid, latissimus dorsi, or biceps brachii were resected due to partial tumor invasion. The rotator cuff, particularly the supraspinatus, infraspinatus, and subscapularis, were excised, similar to the external rotator muscles. Most of the affected muscles surrounding the thoracoscapula required en bloc excision with the tumor. The suprascapular nerve and blood vessel bundle required removal in only one patient (#1). The affected muscles were commonly characterized intraoperatively as swollen, necrotic, and deficient in elasticity/contractibility.

Following excision of affected soft tissues, resection of the acromion base and coracoid process (with preservation of the tips) was performed in all patients. Subsequently, the distal end of the clavicle was resected in patient #2 and the normal glenoid (in patients #2, 3, 6, and 7) was osteomized longitudinally at least 1 cm medial to the glenoid edge in sequence while preserving the glenoid articular capsule and surface (in the glenoid-saved group). Alternatively, for the patients with an involved glenoid (#1, 4, and 5), the glenoid was resected together with the articular surface through an additional incision of the capsule (i.e., the glenoid-resected group). Finally, the affected scapula bodies were resected (in patients #1, 2, 3, 5, and 6) based on analysis of the intraoperative frozen sections that were taken to determine the surgical margins. A wide resection and safe surgical margin was selected for all patients.

### Bone and soft tissue management

The articular capsule and muscles, particularly the abductors, were reconstructed in sequence following bony reconstruction. The fresh-frozen (-80°C) scapular allografts were provided by the bone bank at the authors' medical institution. Size-matched scapula allografts were placed to fit the bone defect, with a posterior glenoid tilt angle of 8° to 12° and a downward slope angle of 4° of the glenoid fossa. Fixtures used for the glenoid-saved allografts depended on the thickness of the remaining glenoid. When the glenoid thickness exceeded 1 cm, the allograft was fixed proximal to the lateral border of the scapula. For patients with a glenoid thickness of less than 1 cm, the articular capsule was instead sutured through holes created at the glenoid edge. The residual scapula were fixed to the glenoid-resected allografts with plates and screws and the articular capsule was sutured circumferentially via holes created in the allograft's glenoid edge. The tip of the coracoid and acrimion were fixed to the corresponding allografts with absorbable screws, sutures, or reconstructive plates to restore the superior suspensory shoulder complex.

Overall, two or more plates were shaped and implanted on the glenoid, spine, or the lateral and medial borders of the scapula according to the size and location of the allografts. These plates were then used to fix the host scapula to the allografts with screws. The resected partial clavicle of one patient (treated with alcohol devitalization) was fixed with a plate to its original position while the distal clavicles of the remaining six patients were bound with Dacron tape.

After implanting the allografts, the abduction mechanism, including the deltoid and rotator cuffs, were reconstructed using the remaining muscles. Posteriorly, deltoid reconstruction was achieved in two patients by tenodesis to the trapezius and intraosseous sutures. The uninvolved deltoid was reattached to its stumps on the allograft, the host acromion process, or the clavicle. The remaining muscles were either sutured to their corresponding stumps or were tenodesed to predrilled holes in the allografts. Rotator cuff reattachment was achieved in four patients. The articular capsule and deltoid were either well preserved and/or reconstructed in all seven patients. Two patients (#3 and 4) required local intraoperative radiotherapy in the muscles surrounding the scapular allograft using I^125^.

### Postoperative rehabilitation programs

The upper extremity was placed in an abduction brace at a functional position for four weeks postoperatively. Range of motion (ROM) and motor strengthening exercises for the hand and elbow were performed immediately postoperatively and shoulder isometric exercises were initiated within five days postoperatively. Later, isotonic and resistance muscle training were included in the patients' rehabilitation programs after removal of the brace.

## Results

**Figure 3:**
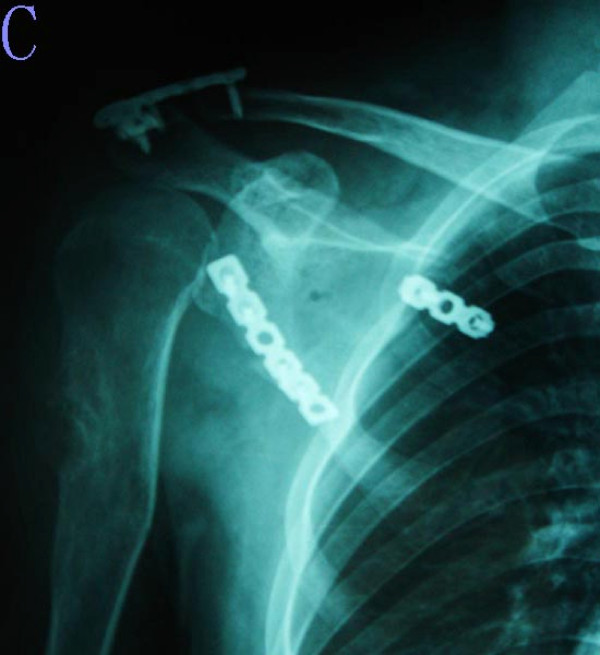
**The postoperative plain radiograph shows the scapular allograft reconstruction**.

**Figure 4:**
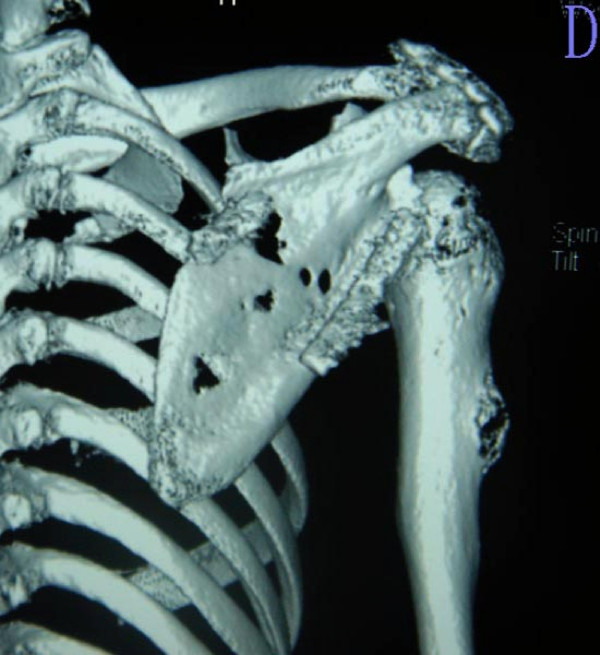
**A 3-D computed tomography reconstruction taken 14 months after the procedure shows satisfactory healing at the host-graft junction together with slight bone resorption**. Dislocation of the shoulder joint and local recurrence is not present.

**Figure 5:**
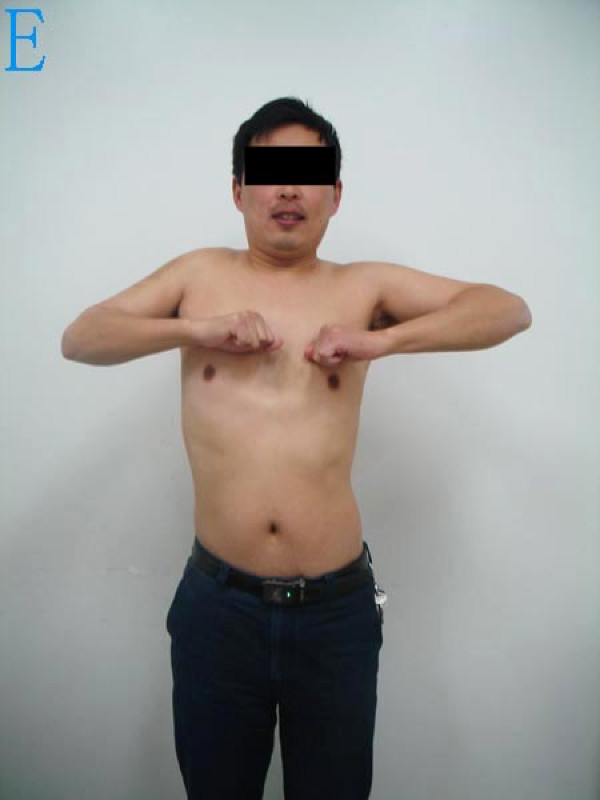
**The shoulder abduction function and appearance 14 months postoperatively**.

The median follow-up period for the seven patients followed in this case series was 26 months (range, 14–50 months). ISOLS-based functional scores ranged from 21 to 28 points (mean, 24) with a mean functional rating of 80% (range, 70–93%). As shown in Table 3, the range of active shoulder abduction and forward flexion motion were 40°–110°and 30°–90°, respectively and all patients retained a high degree of hand and elbow function. Satisfactory shoulder contour was achieved in all patients (Figure 3, Figure 4, Figure 5). Three patients (#4, 6, and 7), whose rotator cuffs were resected, had lower total ISOLS scores (22, 21, and 23 points, respectively) than the other four patients and demonstrated a limited range of shoulder abduction and flexion.

The glenoid-saved reconstruction showed a mean ISOLS score of 24.5 points (81%), compared with 24 points (79%) in the glenoid-resected group of patients; however, the glenoid-saved patients had superior abduction/flexion motion than the glenoid-resected patients (mean, 72°/61° versus 55°/43°). Further, higher scores for emotional acceptance were recorded in the glenoid-saved allograft group than in the glenoid-resected patients. No correlation between the size of the lesion and the degree of postsurgical shoulder function was noted.

Two patients had local recurrence during follow-up. One patient (#6), diagnosed originally with a recurrent aggressive chondroblastoma, had a local recurrence at 28 months postoperatively and died of the disease 36 months after surgery with an intact allograft. Another patient with a preoperative diagnosis of myeloma (#3) was alive at follow-up in spite of the recurrent cancer. One patient (#2) diagnosed preoperatively with chondrosarcoma underwent an additional surgery during the follow-up period due to development of osteochondroma in the proximal humerus. The remaining five patients were alive and tumor-free for the duration of the study follow-up period.

**Figure 6:**
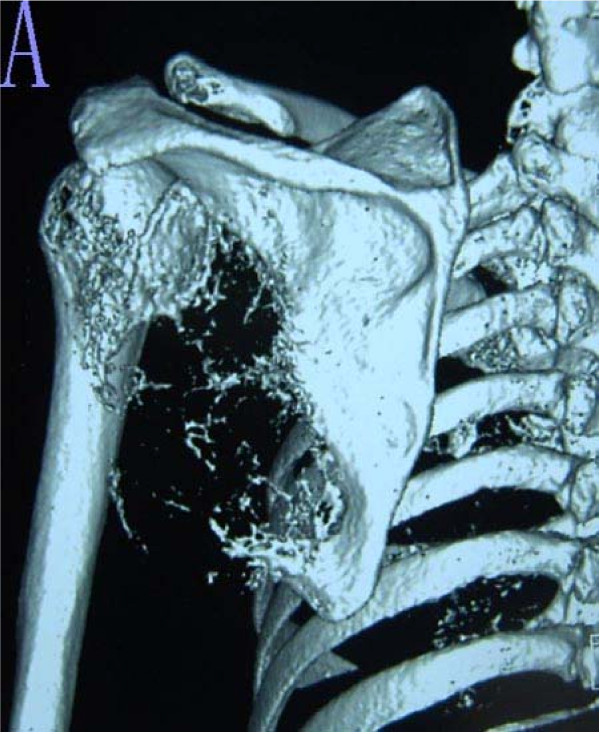
**Radiographs and photograph of the patient with myeloma (#3)**. The plain radiograph shows an expansive lesion in the glenoid, neck, and border of the scapula.

**Figure 7:**
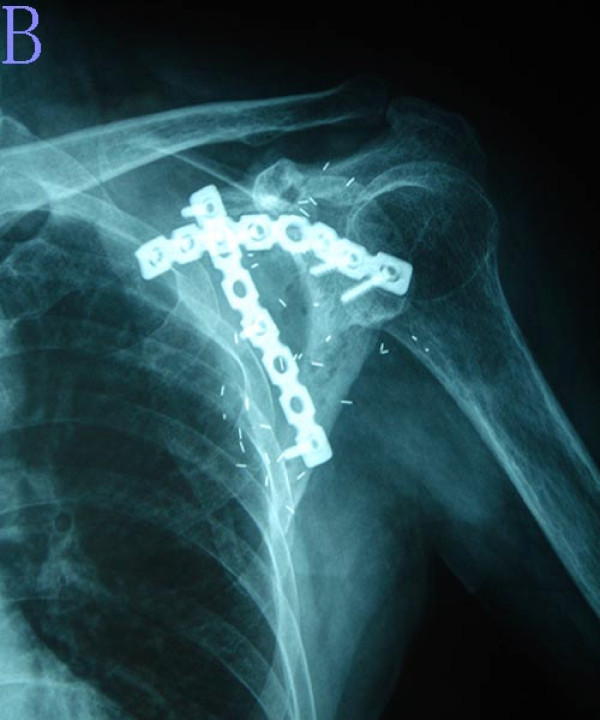
**The plain radiography 20 months after the procedure shows the scapular allograft reconstruction**. The local I^125^ radiotherapy placed around scapular muscles is shown. The union of the scapular allograft is apparent and there is no dislocation of the shoulder joint.

**Figure 8:**
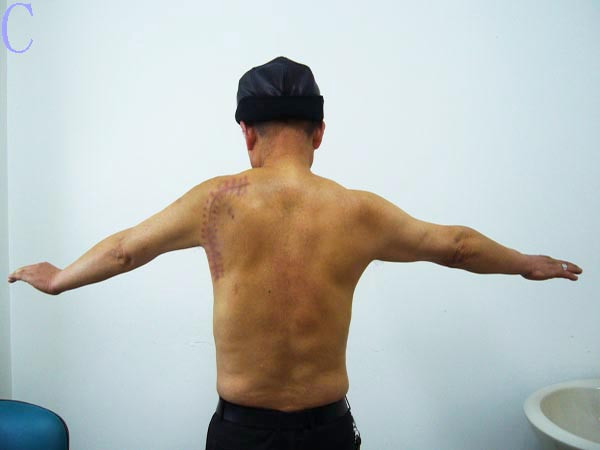
**The acceptable active abduction function and the cosmetic appearance of the left shoulder is shown 20 months postoperatively**.

In terms of postoperative complications, one patient (#2) acquired a deep infection at the distal end of the clavicle, which had been fixed during surgery with a plate. Removal of the plate and surgical debridement was performed 16 months postoperatively, but recovered uneventfully thereafter. Another patient (#4) complained of shoulder pain throughout the follow-up period. There were no nonunions between the allografts and the host scapula, and no shoulder dislocations and articular degeneration were apparent as determined by radiography (Figure 6, Figure 7, Figure 8).

## Discussion

Wide resection and reconstruction of scapular tumors presents a unique surgical challenge requiring an adequate surgical margin while maintaining maximal preservation of the involved soft tissues. In this case series, a preoperative imaging study in conjunction with analysis of intraoperative frozen sections were employed to determine appropriate margins in each patient. The size of the scapular lesion for all seven patients ranged from 5 to 25 cm in length, 4 to 15 cm in width, and 3 to 10 cm in thickness. Bony destruction was commonly detected in region S2 indicating that most patient's lesions exceed or approximated the scapula in median length (155 ± 16 mm) and median width (134 ± 12 mm) as described previously [15]. Furthermore, the soft tissues surrounding the scapula were widely invaded.

The surgical classification system and systemic adjuvant therapy both assist in defining safe resection borders and guiding muscle reconstruction. Type A resections (abductors preserved) and Type I-III resections of the shoulder girdle always entail an intracompartmental resection [14]. Accordingly, partial scapulectomy (Type IIA) and scapular allograft reconstructions were performed successfully in all seven patients described herein.

Chondrosarcomas are primarily located in region S1 (55%) and secondarily in region S2 (23%). Chondrosarcomas in region S1 are treated with partial scapulectomy whereas a total scapulectomy is performed more frequently in patients with a chondrosarcoma larger than 5 cm or for those located in region S2 [16]. This finding is not consistent with the two patients in this series diagnosed with chondrosarcomas (#1 and 2). Instead of a total scapulectomy, a partial scapulectomy was elected for both patients because of the low stage of chondrosarcoma, despite the fact that both tumors were larger than 5 cm and located in region S2. The tumors of the remaining five patients were primarily detected in region S2. The scapular resection for lower stage tumors in these five patients indicated a Type IIA procedure. Among those tumors, chondroblastoma of the scapula is considered an aggressive but benign tumor associated with local recurrence and pulmonary metastasis [17]. Since patient #6 presented with the same features and potential damage as a malignant scapular tumor, we elected to treat this patient with wide resection.

In general, an adequate surgical margin was achieved based on a favorable histological type and surgical stage along with the requisite adjuvant therapy. Therefore, a wide marginal resection that permits the secure reattachment of the important soft tissues of the shoulder should be a therapeutic goal in these patients. Most rotator cuffs, external rotators, and muscles around the thoracoscapula were sacrificed to obtain a safe surgical margin. Nonetheless, we paid particular attention to restoration of essential shoulder abduction, flexion and stability in order to meet out patient' post-operative needs. It should be noted that the relatively intact deltoid and articular capsule are requisite for achieving the desired level of motion and stability.

The initial incision was considered a key factor in obtaining an adequate surgical margin and optimal reconstruction. The incision site and subsequent course was determined with several important goals in mind. One was to expose the bony and muscular elements of the region while providing adequate exposure for allograft reconstruction. Another was to minimize the loss of the uninvolved soft tissue (an opinion which is consistent with other experts in this field [18]). The incision was centered in the middle of each tumor, thus a long posterior/superior approach like an inverted L-shaped incision was predominantly utilized to permit complete exposure of the scapular spine, acromion, and coracoid process and to facilitating internal fixation. Since there was a limitation in exposure for the larger tumors located at the lateral border of the scapula using with this approach, a lateral vertical incision was made for tumors occurring at this location; however, the anterior and posterior deltoid can not be freed or reconstructed easily from this approach.

It should also be noted that the former surgical approach is superior to the later for covering the scapular allografts with a latissimus dorsi flap and facilitating glenoid-saved reconstruction, but if the posterior/superior incision was adopted for tumors located in the lateral border of the scapula, the excessive freed latissimus dorsi flap could be a risk factor for flap necrosis. In addition, the long incision could contribute to an unacceptable scar and the patient's negative emotional response to the surgical outcome. Nonetheless, achieving a safe surgical margin must take priority over cosmetics in these cases.

During allograft reconstruction, internal fixation provides static stability for shoulder joints and attachment sites for soft tissues. Two or more plates can be used to stabilize the scapular allograft on the spine, glenoid, or the lateral and medial border of the scapula thereby achieving equal force distribution on the allograft during shoulder abduction and scapula rotation. The tips of the acromion and coracoid should be preserved which will provide anchor points for the scapular allografts. The attachment sites for muscles and the coracoclavicular ligament should be preserved and the reconstruction of the acromion and coracoid with the bony insertion of the deltoid restores the suspension mechanism of the scapula, securing the stability of glenohumeral joint. The fixation of the clavicle also maintains the effect of clavicle suspension for the shoulder joint. The retroversion angle and downward slope of the glenoid surface should also be an important consideration. As previously reported [15,19], the glenoid tilts at an angle of 8° ± 4° to the posterior and the downward slope of the glenoid has an average angle of 4°. Changes to these angles may result in multidirectional instability or anteroposterior dislocation.

With regard to soft-tissue reconstruction, both the articular capsule and deltoid play important roles in shoulder stability and function. The articular capsule acts as the fulcrum for stabilization of the glenohumeral joint, which, in turn serves as the fulcrum for shoulder abduction. Therefore, the articular capsule requires reconstruction prior to the abductor mechanism in both glenoid-saved and glenoid-resected allograft procedures. The deltoid and supraspinatus muscles are the primary muscles involved in shoulder movement. The deltoid is also the predominant muscle responsible for maintaining stability of the shoulder, suspending the humerus, permitting shoulder abduction, and contributing to the contour of the shoulder. As such, the deltoid requires special attention during reconstruction of the scapular girdle [2,6-9,14]. Wittig et al. [10] also demonstrated the importance of covering the scapula prostheses with a vascularized and functional deltoid. Reconstruction of the residual or uninvolved deltoid also allows for myodesis with the functional trapezius and acts as a potential abductor mechanism. Therefore, the articular capsule, together with the deltoid, provides a dynamic stabilizer for the glenohumeral joint and both structures should be reconstructed whenever possible.

Preservation of both the rotator cuff and deltoid significantly influenced the eventual shoulder abduction capacity in the series of patients described herein. Yasojima et al. [20] demonstrated significant electromyogram activity of the supraspinatus and the middle deltoid during scapular plane abduction. The rotator cuff provides a medially and inferiorly directed force vector on the humeral head, which stabilizes the humeral head against the glenoid [21]. In this study, four patients with adequate rotator cuff reconstruction had significantly better shoulder function compared with the three patients whose rotator cuffs were resected. Thus, it is recommended to preserve the rotator cuffs when possible, as previously suggested [2-4]. Unfortunately, the rotator cuffs, especially the posterosuperior ones, often require resection (as illustrated by the patients included in this case series) making it difficult to preserve the affected rotator cuff while achieving a safe surgical margin. Thus, we suggest that the remaining external rotator can be reattached when the posterosuperior rotator cuff is resected. In patients with a deficient rotator cuff, however, movement of the deltoid should be able to assist in achieving acceptable shoulder function [5]. Therefore, preservation of the deltoid muscle length, when possible, will help increase deltoid moment [22] and maintain shoulder abduction capacity. Additionally, the affected muscle(s) around the thoracoscapular joint is known to be less correlated with stability and function of the glenohumeral joint and does not need to be reattached to obtain thoracoscapular rhythm.

Use of a scapular allograft with satisfactory shoulder function has previously been demonstrated [3,4,12]. The mean ISOLS score reported in this case series was 80% but only 78.5% and 74% in the studies reported by Pritsch and Asavamongkolkul, respectively [8,6]. The glenoid-saved reconstruction technique may better ensure the position and direction of the glenoid and better contribute to the stability of the glenohumoral joint due to the preserved articular capsule. In turn, this is likely a key factor in preventing anteroposterior shoulder dislocation. Furthermore, less articular degeneration is thought to be observed in the glenoid-saved reconstruction compared with the glenoid-resected technique at long-term follow-up. This is due to the fact that the synovia arising from the capsule prevents articular cartilage degeneration.

The low incidence of postsurgical complications, the local tumor recurrence (2 out of 7 patients) and the once case of metastasis (out of 7 patients) were similar to those reported by Mnaymneh [4] and occurred less frequently than patients treated with scapular prostheses [6]. For complications related to scapular allografts such as dislocation, degeneration, and instability of the glenohumeral joint, along with rejection, absorption, nonunions, and deep infections of allografts are primarily observed at follow-up rather than during the immediate postoperative period. In our case series, complications occurred infrequently during the follow-up period. Nonetheless, we hypothesize that complications like articular degeneration and allograft absorption are invariably unavoidable when performing this type of surgery.

## Conclusion

The scapular allograft reconstruction following tumor resection can successfully be performed with satisfactory functional, cosmetic, and oncological results. The glenoid-saved reconstruction is advocated over the glenoid-resected procedure. The deltoid and articular capsule contribute significantly to shoulder function, stability, and contour. Thus, we suggest that their preservation and/or reconstruction is an important consideration during the use of scapular allografts. It is also recommended that the rotator cuff be reconstructed, despite the inherent difficulties associated with its intraoperative reattachment. Though the results presented here demonstrate satisfactory clinical results, the study is limited by short-term follow-up for some patients and the small number of cases. Further research, however, is certainly warranted.

## Competing interests

The authors did not receive any financial assistance from any private organization for this study nor was this study influenced by any financial or non-financial ties to the authors. No patent relating to the content of this manuscript is pending and there is no conflict of interest with any author in relation to the submission and publication of the manuscript.

## Authors' contributions

KZ participated in the collection of clinical data, performed patient follow-ups, and drafted the manuscript. CT made substantial contributions to conception and design of this research and has reviewed the manuscript for important intellectual content and given final approval of the version to be published. HD assisted during patient follow-ups and collection of data. ZX participated in project coordination and assisted with manuscript. Each author has participated sufficiently in this work to take public responsibility for the appropriate portions of the manuscript. All authors read and approve of the final manuscript.
